# Comparison of murine steatohepatitis models identifies a dietary intervention with robust fibrosis, ductular reaction, and rapid progression to cirrhosis and cancer

**DOI:** 10.1152/ajpgi.00041.2019

**Published:** 2019-10-21

**Authors:** Guangyan Wei, Ping An, Kahini A. Vaid, Imad Nasser, Pinzhu Huang, Li Tan, Shuangshuang Zhao, Detlef Schuppan, Yury V. Popov

**Affiliations:** ^1^Department of Liver Surgery, The First Affiliated Hospital of Sun Yat-sen University, Guangzhou, China; ^2^Division of Gastroenterology, Hepatology and Nutrition, Beth Israel Deaconess Medical Center, Harvard Medical School, Boston, Massachusetts; ^3^Division of Gastroenterology and Hepatology, Renmin Hospital of Wuhan University, Wuhan, Hubei, China; ^4^Department of Pathology, Beth Israel Deaconess Medical Center, Harvard Medical School, Boston, Massachusetts; ^5^Department of Colorectal Surgery, The Sixth Affiliated Hospital, Sun Yat-sen University, Guangzhou, China; ^6^Institute of Translational Immunology, University Medical Center, Mainz, Germany

**Keywords:** HCC, hepatocellular carcinoma, HF-CDAA model, NAFLD, NASH

## Abstract

Progressive fibrosis, functional liver failure, and cancer are the central liver-related outcomes of nonalcoholic steatohepatitis (NASH) but notoriously difficult to achieve in mouse models. We performed a direct, quantitative comparison of hepatic fibrosis progression in well-defined methionine- and choline-deficient (MCD) and choline-deficient, amino-acid defined (CDAA) diets with increasing fat content (10–60% by calories) in C57Bl/6J and BALB/cAnNCrl mice. In C57Bl/6J mice, MCD feeding resulted in moderate fibrosis at *week 8* (up to twofold increase in total hepatic collagen content) and progressive weight loss irrespective of dietary fat. In contrast, CDAA-fed mice did not lose weight and developed progressive fibrosis starting from *week 4*. High dietary fat in the CDAA diet model induced the lipid metabolism genes for sterol regulatory element-binding protein and stearoyl-CoA desaturase-2 and increased ductular reaction and fibrosis in a dose-dependent manner. Longitudinal analysis of CDAA with 60% fat (HF-CDAA) feeding revealed pronounced ductular reaction and perisinusoidal bridging fibrosis, with a sevenfold increase of hepatic collagen at *week 12*, which showed limited spontaneous reversibility. At 24 wk, HF-CDAA mice developed signs of cirrhosis with pan-lobular “chicken wire” fibrosis, 10-fold hydroxyproline increase, regenerative nodules, portal hypertension and elevated serum bilirubin and ammonia levels; 80% of mice (8/10) developed multiple glypican-3- and/or glutamine synthetase-positive hepatocellular carcinomas (HCC). High-fat (60%) supplementation of MCD in C57Bl/6J or feeding the HF-CDAA diet fibrosis-prone BALB/cAnNCrl strain failed to result in increased fibrosis. In conclusion, HF-CDAA feeding in C57Bl/6J mice was identified as an optimal model of steatohepatitis with robust fibrosis and ductular proliferations that progress to cirrhosis and HCC within 24 wk. This robust model will aid the testing of interventions and drugs for severe NASH.

**NEW & NOTEWORTHY** Via quantitative comparison of several dietary models, we report HF-CDAA feeding in C57Bl/6 mice as an excellent model recapitulating several key aspects of fibrotic NASH: *1*) robust, poorly reversible liver fibrosis, *2*) prominent ductular reaction, and *3*) progression to cirrhosis, portal hypertension, and liver cancer within 24 wk. High fat dose-dependently activates SREBP2/SCD2 genes and drives liver fibrosis in e HF-CDAA model. These features qualify the model as a robust and practical tool to study mechanisms and novel treatments addressing severe human NASH.

## Graphical abstract

**Figure:**
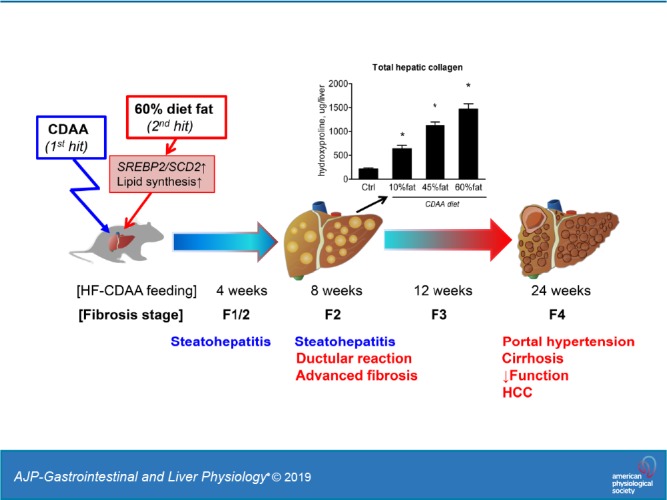

## INTRODUCTION

Nonalcoholic fatty liver disease (NAFLD) has emerged as a growing public health problem, linked to the increased incidence of obesity. Recent studies estimate that between 30 and 40% of the population in the US, 80–100 million Americans, are affected by NAFLD (48, 49). At least 15–20% of NAFLD patients develop nonalcoholic steatohepatitis (NASH), the aggressive form of NAFLD that is characterized by inflammation and fibrosis. NASH can progress to cirrhosis and/or hepatocellular carcinoma (HCC) and in the post-hepatitis C virus era has become the leading cause of liver-related morbidity and mortality. With the aging of the population and increased duration of exposure to the disease, most models predict that the prevalence of NAFLD-related cirrhosis and end-stage liver disease is going to increase dramatically (10). Thus, there is an urgent need to develop new, effective pharmacological therapies for NASH/NAFLD. Because of the innate limitations in acquiring human liver tissue for ex vivo drug testing and the lack of whole body complexity in studying, e.g., precision-cut liver slices (28), development and validation of robust preclinical disease models that mimic clinically important aspects of human NAFLD/NASH is central to mechanistic studies and successful drug development (11).

Several recent studies have firmly established that liver fibrosis stage is a main determinant of liver-related morbidity/mortality in human NASH (1, 9, 14, 43). However, the particular disease aspect of progressive liver fibrosis has been challenging to recapitulate in NASH models, particularly in mouse systems. A number of dietary mouse models of NASH have been reported, with varying degrees of fidelity to metabolic profile and histological signs seen in human NASH (13, 41). Of note, diet-induced obesity (“metabolically faithful” models of overfeeding on high-fat diet) results in simple steatosis, mild steatohepatitis and no fibrosis; this prompted the development of the “Western diet” approach, a combination of a high-fat and -fructose diet combined with supranormal amounts of cholesterol (up to 2%) that results in steatohepatitis. However, even this aggressive dietary intervention results in a relatively mild, slowly progressing fibrosis, requiring up to 6 mo for advanced fibrosis and 12 mo for cirrhosis to develop (2, 5). Practically, this represents a serious caveat for mechanistic studies and rigorous testing of novel NASH drug candidates, especially for liver fibrosis/cirrhosis.

The susceptibility to liver fibrosis also varies greatly depending on species and on the genetic makeup within a particular species. This has been extensively documented in hepatotoxin-induced injury models in mice, where common inbred strains demonstrate vast differences in the degree of liver scarring, ranging from complete resistance in FVB mice to high susceptibility in BALB/c mice (15, 17, 45), underscoring the importance of careful genetic background selection to achieve progressive fibrosis.

Another important disease aspect that is rather poorly captured in animal models is progression to cirrhosis and its sequelae, such as HCC, a growing concern in NAFLD (50). Moreover, although progression to cirrhosis in human NASH is strongly associated with cholangiocyte proliferation, i.e., the ductular reaction (37), no consistent data on the extent of the ductular reaction across mouse models of NASH exist. Moreover, incidence, burden, and phenotype of HCC in NASH models are typically neither rigorously characterized nor used as an end point in preclinical studies. Thus, there is a clear need of a better characterization of NASH models coupled with establishment of standard methodology to assess the effect of treatment on long-term outcomes such as cirrhosis and the tumor burden (11).

There has been very little systematic effort to compare NASH models using robust quantitative methodology as well as phenotypic characterization of inbred mouse strains subjected to even a single model. This makes rational selection of a NASH model for preclinical research or drug evaluation a challenging task, especially for the assessment of true fibrotic pathways and antifibrotic effects. Two recent reports suggested that an increased fat content in commonly used methionine- and choline-deficient diet (MCD) and choline-deficient, amino-acid defined (CDAA) diets can promote fibrosis in mice (6, 24). This prompted us to perform an in-depth quantitative analysis focusing on the role of dietary fat on extent of fibrosis, the ductular reaction, and the hard end points, i.e., cirrhosis and HCC, in the MCD as well as the CDAA model, carried over several periods of time and two common mouse strains.

Here, we have identified the HF-CDAA diet in C57Bl/6J mice as an optimal and practical model of steatohepatitis, with a robust fibrosis and ductular reaction driven primarily by high dietary fat intake. Longitudinal analysis of HF-CDAA-fed mice has further demonstrated rapid progression to clinically significant outcomes of cirrhosis and primary liver cancer.

## MATERIALS AND METHODS

### 

#### Animal experiments.

Male C57BL/6J (no. 000644) inbred mice were purchased from Jackson Laboratories (Bar Harbor, ME); the highly fibrosis-susceptible substrain, BALB/cAnNCrl (stock no. 028) mice, were obtained from Charles River (Wilmington, MA). All mice were acclimatized for 1 wk before experiments and housed on a 12:12 h dark-light cycle and fed a standard rat chow and tap water ad libitum. Animal experiments were approved by the Institutional Animal Care and use Committee [Beth Israel Deaconess Medical Center (BIDMC); protocol nos. 158-2008 and 004-2012) and were conducted in the same facility room at BIDMC.

#### Mouse model of nonalcoholic steatohepatitis (NASH).

All diets were formulated and manufactured by Research Diets (New Brunswick, NJ), and their detailed nutritional composition is reported in Supplemental Table S1 (supplemental material for this article is available online at https://doi.org/10.6084/m9.figshare.7692398.v2. Importantly, all major dietary components of synthetic diets are well defined. Eight-week-old male C57Bl/6J (Jackson Laboratory) or BALB/c mice (Charles River) were fed the MCD diet supplemented with normal (A02082002B) or high fat (60% kcal as fat, A06071301B) for up to 8 wk. The choline-deficient, amino acid-defined diet (CDAA) contained 10% (LF-CDAA, A06071324), 45% (A06071323) and 60% (HF-CDAA, A06071302) fat by calories and was formulated to be matched calorie-wise by sucrose/fructose content. Diets were administered to 8-wk-old male C57Bl/6J mice or BALB/c mice for up to 24 wk. All diets were obtained from Research Diets. Regular chow (Purina, 5008) was used as normal diet control.

#### Hepatic hydroxyproline determination.

Hepatic collagen content was determined as relative hydroxyproline (µg/g liver) in 100- to 200-mg liver samples from two different lobes (representing >10% of whole liver) after hydrolysis in 6 N HCl for 16 h at 110°C, as described (31). Total hydroxyproline (mg/whole liver) was calculated based on individual liver weights and the corresponding relative hydroxyproline content (31, 32).

#### Fibrotic matrix stability assessment.

Fibrotic matrix stability was assessed biochemically ex vivo by complete collagen fractionation through serial extractions as previously described in detail (23). Briefly, 500 mg of snap-frozen tissue from two liver lobes was homogenized, and a series of overnight extractions (1:20, wt/vol) under increasingly harsh conditions was performed to obtain the following collagen-containing fractions: acetic acid soluble (non-cross-linked collagens and procollagens), pepsin soluble (fibrillar, mature, and moderately cross-linked collagens), and insoluble (the remaining, highly cross-linked collagens). Collagen content in each fraction was quantified via hydroxyproline determination after complete acidic hydrolysis and expressed as percentage of hydroxyproline recovered in all fractions.

#### Histology, immunohistochemistry, and immunofluorescence.

Connective tissue stain (Sirius red), hematoxylin-eosin (HE), and immunohistochemistry (IHC) were performed in formalin-fixed, paraffin-embedded liver sections or snap-frozen liver pieces, as described previously (30). Detailed information about antibodies used is summarized in Supplemental Table S2.

#### Liver tumor immunotyping and tumor burden assessment.

All macroscopically visible tumor lesions larger than 2 mm were formalin fixed, embedded, sectioned, and analyzed histopathologically by an experienced pathologist (I. Nasser). IHC staining for the HCC markers glutamine synthetase (GS) and glypican-3 was performed as previously described (40). Glypican-3 staining was considered positive when a moderate to strong nuclear, cytoplasmic, and/or membranous staining was seen in at least 10% of cells. Glutamine synthetase (GS) was considered positive if moderate to strong diffuse staining was observed in at least 50% of cells. Tumors positive for GS, glypican-3, or both markers were classified as HCC cells. Tumors were additionally characterized by reticulin and Ki-67 stainings performed as described (17, 21).

#### Portal venous pressure measurement.

Mice were anesthetized with isoflurane (1.5% vol/vol) via precise vaporizer. After laparotomy, the portal vein was cannulated, and portal pressure was measured directly by inserting a 1.2-Fr high-fidelity pressure catheter (Scisense, London, ON, Canada). Pressure signals were recorded at 2 kHz for 5 min and analyzed using PowerLab software (ADInstruments, Colorado Springs, CO), as described previously (17).

#### Serum biochemistry.

Serum levels of alanine aminotransferase (ALT), total bilirubin (TBIL), and ammonia (NH_3_) were measured using an automated Catalyst Dx Chemistry Analyzer (IDEXX Laboratories, Westbrook, ME) according to the manufacturer’s recommendations.

#### Statistical analyses.

Data are expressed as means ± SE, and statistical analyses were performed using Microsoft EXCEL and GraphPad Prism version 5.00 (GraphPad Software, San Diego, CA). Multiple comparisons were performed by one-way analysis of variance (ANOVA). *P* values < 0.05 were considered significant.

Additional supplementary material can be found via the following link: https://doi.org/10.6084/m9.figshare.7692398.v2.

## RESULTS

### 

#### Direct comparison of fibrotic response in mouse livers after 8 wk of MCD or CDAA diets with low or high fat content.

**Fig. 1.**
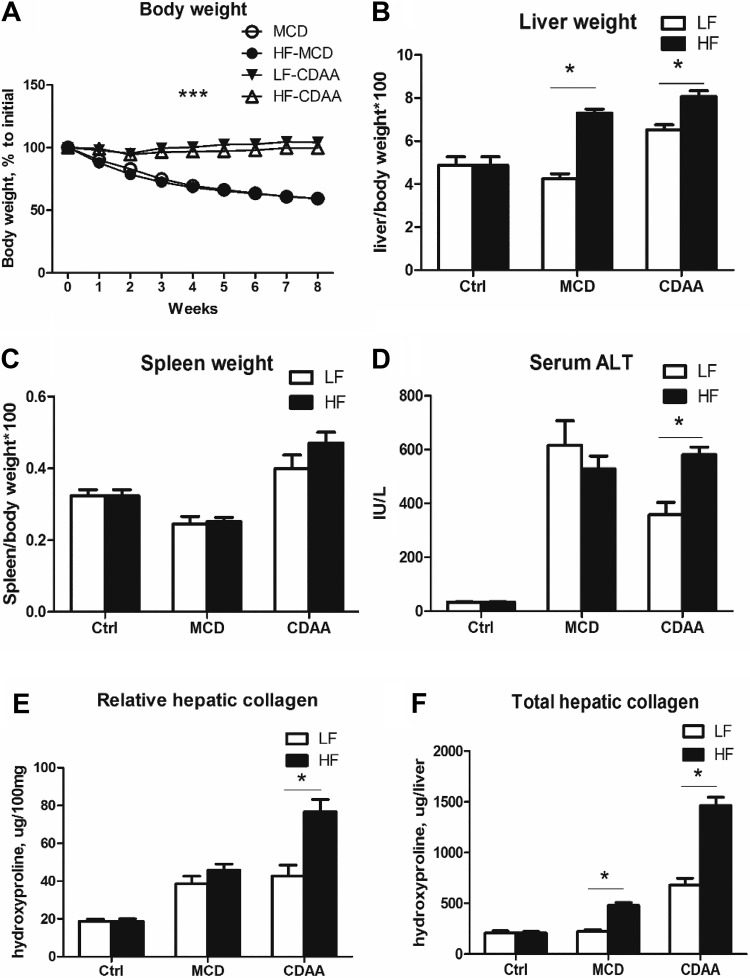
Direct comparison of advanced liver disease phenotype induced by 8 wk of methionine- and choline-deficient (MCD) and choline-deficient, amino acid-defined (CDAA) diets with low (LF) or high fat (HF) content in C57Bl/6J mice. *A*: body weight dynamics show a gradual and significant weight loss on MCD but not CDAA diet irrespective of dietary fat content. Relative liver (*B*) and spleen (*C*) weights (normalized to body weight). *D*: serum alanine aminotransferase (ALT) levels. Relative (per 100 mg tissue; *E*) and total (per whole liver; *F*) hepatic collagen content determined biochemically via hydroxyproline. Data are means ± SE; *n* = 7–10 mice per group. *P < 0.05 vs. controls on normal diet, ***P < 0.001 (two-way ANOVA followed by Bonferroni posttest).

Diet, food intake patterns, and diet composition are critical factors for NAFLD development, but the impact of these nutritional parameters on fibrotic responses in various animal models has not been well characterized. Here, we analyzed the extent of liver fibrosis biochemically (using the “gold standard” hydroxyproline measurement) and histologically (using Sirius red morphometry), in diet-induced obesity-prone C57Bl/6J mice fed MCD or CDAA diets for 8 wk, with either low fat (LF, 10% of calories), or high fat (HF, 60% of calories) content. In agreement with published reports (6, 24), mice on the MCD, but not on the CDAA diet, developed a marked weight loss that did not permit MCD feeding past 8 wk. Interestingly, the HF diet did not attenuate the weight loss (Fig. 1*A*). However, the HF diet promoted hepatomegaly in both the MCD and CDAA groups, with 72 and 24% increase in liver relative to body weight compared with the LF-MCD and LF-CDAA diets, respectively (Fig. 1*B*), whereas there was no significant difference in spleen weights (Fig. 1*C* and Supplemental Table S3). Liver injury, as assessed via serum ALT levels, was highly increased and did not differ between LF-MCD, HF-MCD, and HF-CDAA groups but appeared to be considerably lower in the LF-CDAA group (Fig. 1*D*). In the MCD diet-fed mice, biochemical methods showed moderate (2-fold vs. controls) increases in relative hydroxyproline content (a measure of collagen content per 100 mg of liver tissue) in both the LF-MCD-fed and HF-MCD-fed mice (Fig. 1*E*); however, total hepatic hydroxyproline (a measure of collagen content per whole liver) was increased only twofold in HF-MCD compared with LF-MCD mice or healthy controls (Fig. 1*F*). This was due to the increase in liver volume due to HF supplementation (Fig. 1*B*). Mice fed the LF-CDAA diet demonstrated increases of relative hydroxyproline content similar to those of the MCD diet-fed mice; however, addition of high fat to CDAA (60%, HF-CDAA) markedly increased collagen deposition (4-fold vs. healthy controls and 2-fold vs. the LF-CDAA group; Fig. 1*E*). Increases in total liver hydroxyproline content in the HF-CDAA mice were even more significant (7-fold vs. normal controls, *P* < 0.001; Fig. 1*F* and Supplemental Table S3). This was accompanied by severalfold higher expression levels of profibrogenic genes in HF-CDAA- vs. HF-MCD diet-fed mice (Supplemental Fig. S1). Thus, HF-CDAA diet feeding in C57Bl/6J mice resulted in remarkably robust fibrosis, using objective, quantitative biochemical methods, without the body weight loss observed in MCD diet-fed mice. Steatohepatitis progression on the LF/HF-MCD diets was also characterized longitudinally at 2-, 4-, and 8-wk time points and followed the expected course based on the 8-wk end point data (see Supplemental Table S4 and Supplemental Figs. S1 and S2 for details). Because C57Bl/6J mice have a limited susceptibility to fibrosis in models of postnecrotic hepatic fibrosis, such as models based on the hepatotoxins carbon tetrachloride (15) or thioacetamide (17) compared with high susceptibility in the hepatic fibrosis-prone strain BALB/cAnNCrl (17), we explored whether the fibrotic response can be further boosted by subjecting BALB/cAnNCrl mice to HF-CDAA or HF-MCD feeding. Surprisingly, we did not observe significant differences in relative or total hepatic collagen levels, despite similar liver injury (as assessed via serum alanine transferase, ALT) between the BALB/c and C57Bl/6J strains fed the low or high fat MCD diets for 8 wk (Supplemental Fig. S3 and Supplemental Table S5). In addition, HF-CDAA feeding caused a comparable liver injury (ALT levels) in both mouse strains, but increases in both relative and total hepatic collagen were moderately but significantly lower in BALB/c compared with C57Bl/6J mice at *week 8* (Supplemental Fig. S3). Histologically, connective tissue staining showed a similar pattern of fibrosis in both strains, with the extent of fibrosis generally being consistent with the biochemical data on hepatic collagen deposition (Supplemental Fig. S3).

#### Histological characterization of hepatic stellate cell activation and the ductular reaction in LF- or HF-MCD and CDAA diets.

**Fig. 2.**
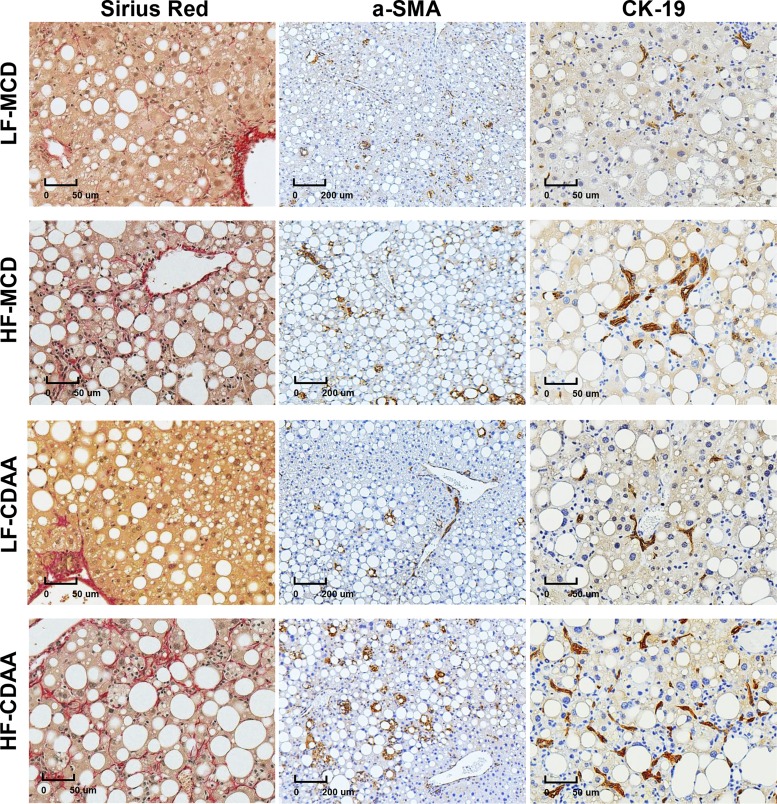
Representative histopathology after 8 wk of methionine- and choline-deficient (MCD) and choline-deficient, amino acid-defined (CDAA) diets with low (LF) or high-fat (HF) content in C57Bl/6J mice. Representative images of connective tissue staining (Sirius red, *left*, ×200), and immunohistochemistry (IHC) for hepatic stellate cell (HSC) activation marker α-smooth muscle actin (α-SMA, *middle*, ×50; *B*), and the ductular reaction visualized via cytokeratin 19 IHC (CK19, *right*, ×200; *C*). Note the remarkable, pan-lobular expansion of CK19+ cells accompanied by increased HSC activation in HF-CDAA livers.

Next, we performed a direct comparison of the histological features of liver inflammation and fibrosis after 8 wk of the MCD and CDAA diets with low or high fat content in C57Bl/6J mice. Consistent with biochemical collagen quantification, all groups developed various degrees of histologically apparent perisinusoidal fibrosis visualized by collagen (Sirius red) staining. Accordingly, all HF-CDAA livers demonstrated remarkably robust sinusoidal fibrosis at 8 wk, by far exceeding the scarring observed in all other treatment groups (Fig. 2). Both hepatic stellated cell activation (α-SMA IHC) and the ductular reaction (CK19 IHC labeling, apart from mature bile ducts, reactive cholangiocytes and hepatic progenitor cells) were modest in the MCD or CDAA mice on the LF diet but increased markedly in mice on the HF diet. Increases in α-SMA-positive cell numbers and pan-lobular expansion of CK19+ cells were particularly remarkable in HF-CDAA livers, consistent with the more severe fibrosis in this group (Fig. 2).

#### Longitudinal analysis reveals robust and progressive liver fibrosis induced by HF-CDAA diet.

**Fig. 3.**
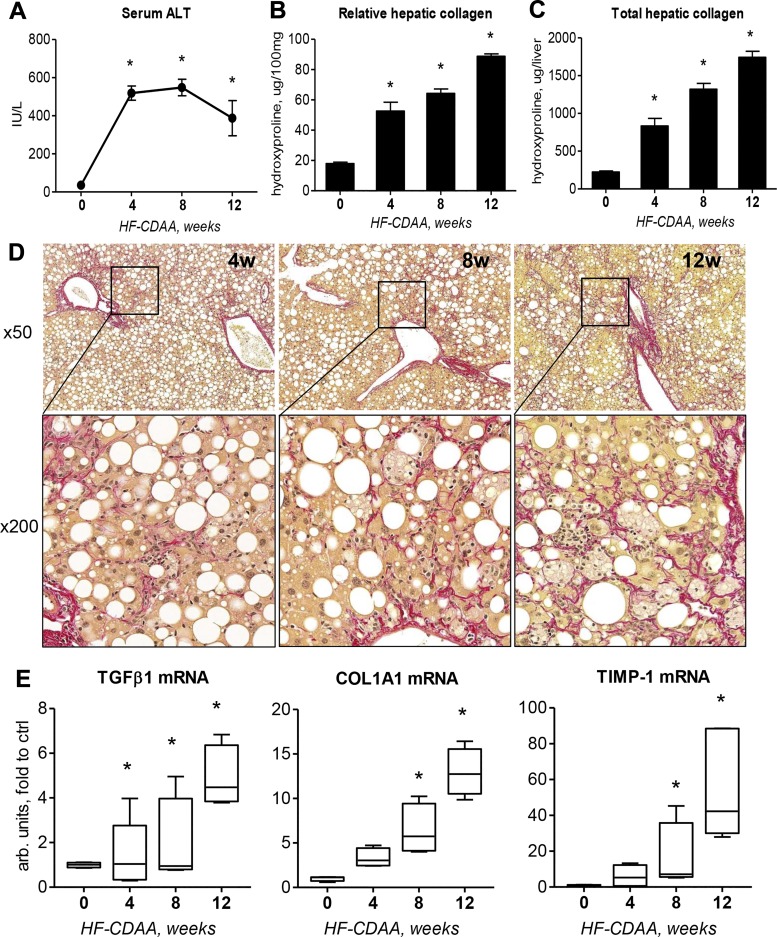
Progression of steatohepatitis induced by the 60% high-fat (HF) choline-deficient, amino acid-defined (CDAA) diet. C57Bl/6J mice were fed HF-CDAA diet ad libitum for 4, 8, and 12 wk (*n* = 5 per time point; the 0-wk group represents age-matched healthy control mice fed standard chow). *A*: serum alanine aminotransferase (ALT) levels. Relative (per 100 mg tissue; *B*) and total (per whole liver; *C*) hepatic collagen content determined biochemically via hydroxyproline. *D*: representative low- (*top*, ×50) and high- (*bottom*, ×200) magnification images of collagen staining (Sirius red) demonstrate robust perisinusoidal fibrosis starting from *week 4*, progressing to advanced perisinusoidal fibrosis at *week 8* and to severe pan-lobular “chicken wire” fibrosis at *week 12*. *E*: pro-fibrogenic transcript levels of transforming growth factor-β1 (TGFβ1), procollagen α1(I), and tissue inhibitor of metalloproteinases 1 (TIMP-1) in livers of HF-CDAA-fed mice. Col1A1, collagen 1A1. Data are means ± SE. *P < 0.05 vs. healthy controls (one-way ANOVA followed by Dunnett’s posttest).

To characterize the longitudinal liver fibrosis progression, 8-wk-old C57Bl/6J male mice were fed the HF-CDAA diet for 4, 8, and 12 wk. These mice did not develop any weight loss or emaciation, as observed in the MCD-fed mice. Serum ALT was significantly elevated at *weeks 4* and *8* and declined at *week 12*, still well above normal levels (387.60 ± 92.35 vs. 35.40 ± 2.98 in chow-fed controls at *week 0*, *P* = 0.0051; Fig. 3*A*). Biochemically, both relative and total hepatic collagen levels significantly and progressively increased from *week 4* through *week 12* (Fig. 3, *B* and *C*, and Supplemental Table S8). At 12 wk, relative hydroxyproline was increased more than fourfold compared with healthy controls (88.80 ± 1.58 vs. 17.93 ± 0.92, *P* < 0.0001; Fig. 3*B*), and total hepatic collagen increased nearly eightfold (1,742.00 ± 79.56 vs. 223.80 ± 13.76, *P* < 0.0001; Fig. 3*C*). Histologically, connective tissue staining revealed readily detectable perisinusoidal fibrosis starting at week 4 and progressing to severe pan-lobular “chicken wire” fibrosis occupying most of the liver parenchyma at *week 12* (Fig. 3*D*). Fibrosis in all livers was staged by an experienced pathologist (I. Nasser) in a blinded manner according to the NASH CRN scoring system (19). HF-CDAA diet feeding induced a moderate fibrosis (F1/2) in all mice at *week 4*, progressing to prominent periportal-sinusoidal fibrosis (F2) at *week 8*, and to bridging pan-lobular fibrosis with complete septae (F3) at *week 12*. Finally, hepatic expression of the key profibrogenic transcripts transforming growth factor-β1 (*tgfb1*), procollagen type 1 (c*ol1a1*), and tissue inhibitor of metalloproteinases 1 (*timp1*) were progressively and massively upregulated up to 5, 10, and 28 times above that of healthy control levels, respectively (Fig. 3*E*).

#### High dietary fat dose-dependently promotes ductular reaction and liver fibrosis progression in the CDAA model.

**Fig. 4.**
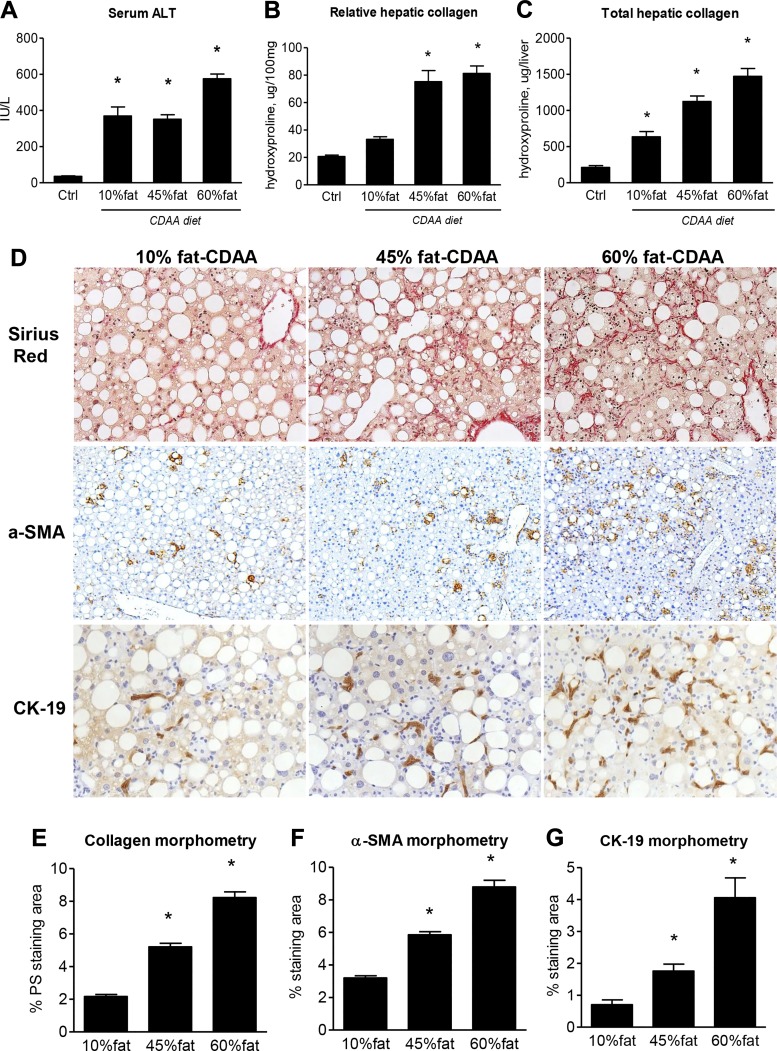
Dietary fat dose-dependently promotes the ductular reaction, hepatic stellate cell (HSC) activation, and liver fibrosis progression in the choline-deficient, amino acid-defined (CDAA) diet induced steatohepatitis model in C57Bl/6J mice. *A*: serum alanine aminotransferase (ALT) levels. Relative (per 100 mg tissue; *B*) and total (per whole liver; *C*) hepatic collagen content. *D*: connective tissue staining (Sirius red; *top*, original magnification ×200), immunohistochemistry for HSC activation marker (α-SMA; *middle*, original magnification ×50), and ductular reaction marker [cytokeratin 19 (CK19); *bottom*, original magnification, ×200]. Morphometric quantification of collagen staining (*E*)-, α-SMA (*F*)-, and CK19 (*G*)-positive area. Data are means ± SE; *n* = 9–10 mice per group. *P < 0.05 vs. healthy controls on regular chow; n.s., not significantly different (one-way ANOVA followed by Dunnett’s posttest).

To further characterize the impact of dietary fat content on the fibrotic responses in the CDAA model, 8-wk-old C57Bl/6J male mice were fed the CDAA diet with increasing proportions of fat (10, 45, and 60% by calories) for 8 wk. HF supplementation of CDAA diet did not lead to overnutrition, with daily food intake (calorie-wise) in the 60% fat-CDAA diet fed C57Bl6J mice remaining constant throughout the 8 wk of feeding, and virtually identical to reference values reported for standard chow in this strain (11.81 ± 0.05 vs. 12.12 ± 0.23 kcal per mouse per day in normal chow-fed C57Bl/6, according to Bachmanov et al. (3). Liver injury, as determined via serum ALT, was increased similarly in the 10 and 45% fat CDAA groups, and further elevated in the 60% fat CDAA mice (Fig. 4*A*). In contrast, liver fibrosis was dose-dependently impacted by the dietary fat content, as assessed by both biochemical and histological methods. Thus, compared with healthy controls, relative collagen content was increased 1.65-, 3.75- (*P* < 0.0005), and 4.05-fold (*P* < 0.0001), and total hepatic collagen content was increased 3.0- (*P* = 0.0013), 5.3- (*P* < 0.0001), and 6.9-fold (*P* < 0.0001) in the 10, 45, and 60% fat CDAA groups, respectively (Fig. 4, *B* and *C*, and Supplemental Table S9). Histologically, connective tissue staining revealed minimal sinusoidal fibrosis in the 10% fat group, intermediate sinusoidal fibrosis in the 45% group, and advanced “chicken wire” pan-lobular fibrosis in the 60% fat CDAA group (Fig. 4*D*, *top*). Morphometry revealed a 2.5- and 4-fold increase in collagen area in the 45 and 60% fat CDAA groups, respectively, compared with the 10% fat group (Fig. 4*E*). Because human NASH progression correlates strongly with the extent of the ductular reaction (37), we performed immunostainings for markers of the ductular reaction (CK19) and hepatic stellate cell activation (α-SMA), which revealed that both ductular reaction and hepatic stellate cell activation were promoted by dietary fat in a dose-dependent fashion (Fig. 4*D*). Quantitative morphometry demonstrated that α-SMA+ cells were 1.83-fold and 3-fold increased in the 45 and 60% fat CDAA groups, respectively, compared with the 10% fat CDAA group (Fig. 4*F*). This was paralleled by an up to fourfold increase in CK19+ cells in the 60% fat vs. the 10% fat CDAA group (Fig. 4*G*). Together, these results suggest that dietary fat intake dose-dependently drives liver fibrosis progression in the CDAA-induced NASH model via a mechanism that appears to be linked to a pronounced profibrogenic ductular reaction, similarly to that in human progressive NASH.

#### HF supplementation of CDAA diet selectively activates genes regulating monounsaturated fatty acid biosynthesis.

**Fig. 5.**
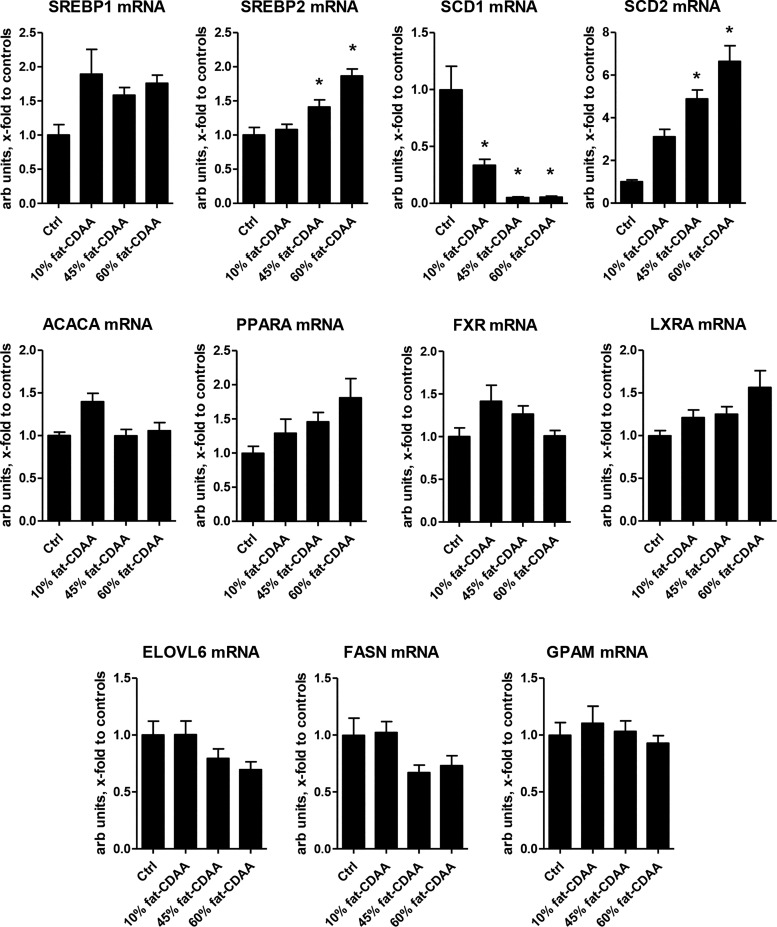
Hepatic lipid metabolism-related gene expression in C57Bl/6J mice fed choline-deficient, amino acid-defined (CDAA) diets with increasing fat content. Key lipid metabolism-related transcripts were quantified by qRT-PCR from total liver RNAs: sterol regulatory element-binding protein-1 and -2 (SREBP1, SREBP2); stearoyl-CoA desaturase-1 and -2 (SCD1, SCD2); acetyl-CoA carboxylase-1 (ACACA); peroxisome proliferator-activated receptor-α (PPARA); farnesoid X receptor (FXR); liver X receptor-α (LXRA); elongation of very long-chain fatty acid protein-6 (ELOVL6); fatty acid synthase (FASN); and glycerol-3-phosphate acyltransferase-1 mitochondrial (GPAM). Data are means ± SE; *n* = 4–10 mice per group expressed as fold to healthy controls and relative to β_2_-microglobulon (b2MG) as housekeeping gene. *P < 0.05 vs. healthy controls on regular chow (one-way ANOVA followed by Dunnett’s posttest).

To characterize hepatic lipid metabolism-related changes underlying profibrogenic effect of high dietary fat, we surveyed hepatic expression of 11 key lipid metabolism genes (see Supplemental Table S6 for the complete list and functional annotation) via qRT-PCR in C57Bl/6J mice fed ghe CDAA diet with increasing proportions of fat (10, 45, or 60% by calories) for 8 wk. Increasing fat dose-dependently and selectively impacted genes responsible for monounsaturated fatty acid biosynthesis (SREBP2 and SCD1 and SCD2) in the context of CDAA feeding, whereas other genes remained largely unaffected (Fig. 5). SCD1 was downregulated threefold by CDAA diet feeding and further suppressed ~18-fold by both HF regimens (45 and 60%) compared with normal chow controls (*P* < 0.0001, ANOVA). In contrast, SCD2 was dose-dependently induced by dietary fat up to 6.6-fold in the 60% fat-CDAA diet-fed livers versus normal chow controls (*P* < 0.0001, ANOVA). In parallel, SREBP2 (but not SREBP1) was dose-dependently increased by dietary fat up to twofold in 60% fat-CDAA-fed mice vs. chow controls (*P* < 0.0001, ANOVA; Fig. 5). Some other lipid metabolism-related genes showed only a trend of downregulation (farnesoid X receptor, fatty acid synthase, elongation of very long-chain fatty acid-like), or upregulation (peroxisome proliferator-activated receptor-α, liver X receptor) in response to increased dietary fat content, not reaching statistical significance in multivariate analysis (*P* > 0.05, ANOVA). When we compared strain-specific differences in hepatic lipid metabolism-related profiles (from the experiments described in Supplemental Fig. S3 and Supplemental Table S5), the same set of genes was significantly impacted in response to 60% fat-CDAA in BALB/c: i.e., SCD1 was downregulated to the same extent as in C57Bl/6J [*P* = 0.6791, BALB/c vs, C57Bl/6J, not significant (NS)], whereas SCD2 and SREBP2 were induced to a considerably lesser degree (*P* = 0.011 and *P =* 0.037, respectively), consistent with an attenuated fibrotic response in this model (BALB/c < C57Bl/6; Supplemental Table S7 and Supplemental Fig. S3).

#### Limited reversibility of advanced liver fibrosis induced by HF-CDAA feeding.

**Fig. 6.**
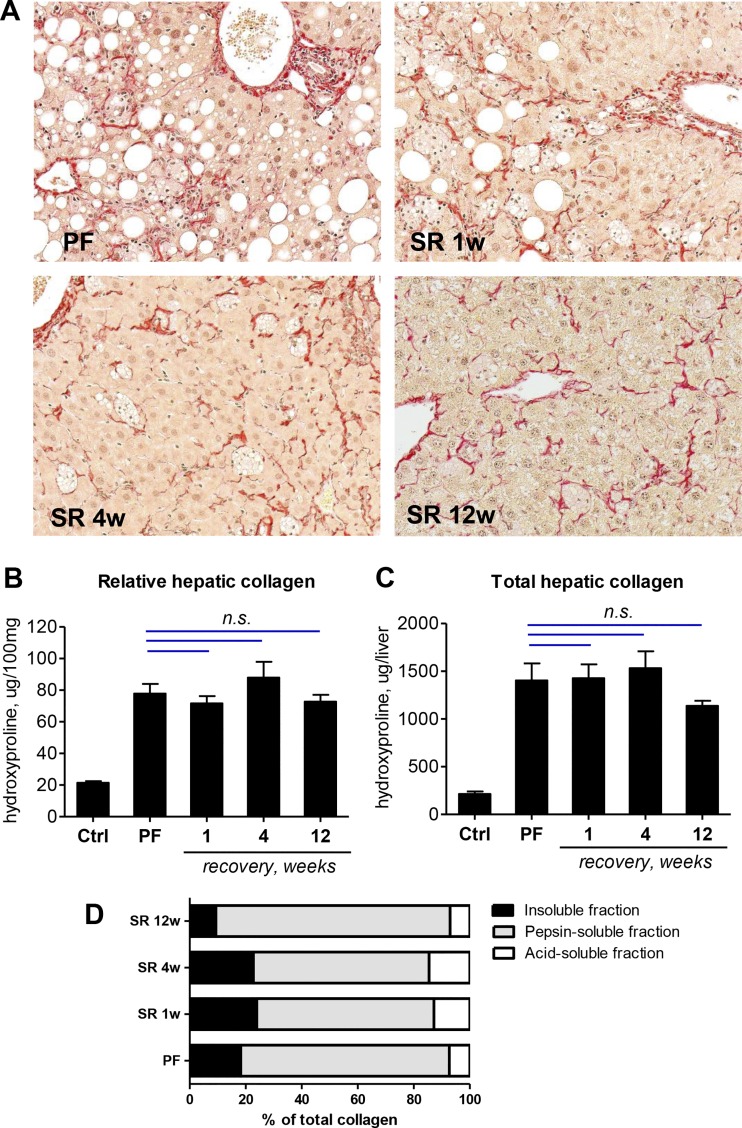
Limited reversibility of advanced liver fibrosis induced by high-fat choline-deficient, amino acid-defined (HF-CDAA) diet feeding. C57Bl/6J mice were fed the HF-CDAA diet for 8 wk (PF, peak fibrosis), switched to a regular chow, and were allowed to recover for 1, 4, and 12 wk (SR, spontaneous recovery). *A*: collagen staining (Sirius red, original magnification ×200) shows complete reversal of steatosis and significant architectural remodeling by *week 4* but persistence of perisinusoidal collagen deposits even at 12 wk of recovery. No significant changes observed in relative (*B*) and total hepatic collagen content (*C*) for up to 12 wk of recovery. *D*: fibrotic matrix stability at PF and throughout recovery, assessed ex vivo by collagen fractionation assay as described in materials and methods. Data are means ± SE; *n* = 5 per bar; n.s., not significantly different (*P* > 0.05) vs. PF group (ANOVA followed by Dunnett’s posttest).

Next, we assessed the extent to which HF-CDAA-induced liver fibrosis is reversible. We induced advanced fibrosis in C57Bl/6J mice by 8 wk of HF-CDAA diet feeding (PF, peak fibrosis), after which fibrotic mice were switched to regular chow to allow spontaneous recovery (SR) for 1, 4, or 12 wk. Histologically, connective tissue staining demonstrated that HF-CDAA livers underwent rapid and remarkable reversal of hepatic steatosis and inflammation accompanied by substantial tissue remodeling. Steatosis visibly improved after 1 wk of recovery and disappeared by *week 4*; immune cell infiltrates persisted for up to 4 wk and disappeared by *week 12*. The fibrotic scar also underwent dramatic remodeling, with characteristic (36) signs such as the splitting of fibrotic bands that became apparent at *week 4* of recovery. However, collagen fibers continued to persist, being loosely spread throughout the liver parenchyma even after 12 wk of recovery (Fig. 6*A*). Biochemically, both relative and total hepatic collagen content remained unchanged throughout recovery compared with the “PF” group (*P* > 0.05, ANOVA), indicating that no significant collagen degradation and removal occurred during spontaneous recovery despite changes in fibril diameter and distribution (Fig. 6, *B* and *C*, and Supplemental Table S10). Direct biochemical analysis of the fibrotic matrix using a stepwise collagen extraction assay (36) in HF-CDAA diet-fed mice revealed that more than 18% of collagens at PF was present in the highly cross-linked (insoluble) fraction. Moreover, collagen cross-linking continued throughout 4 wk of recovery after switching of the HF-CDAA diet to regular chow (22.76 ± 1.24% in the insoluble fraction vs. 18.24 ± 1.25% in the PF group, *P* = 0.0334, *t* test; Fig. 6*D*), before declining at 12 wk of recovery (9.35 ± 3.02% vs. 18.24 ± 1.25% in the PF group, *P* = 0.0263, *t* test).

#### Long-term HF-CDAA feeding leads to development of cirrhosis and HCC.

**Fig. 7.**
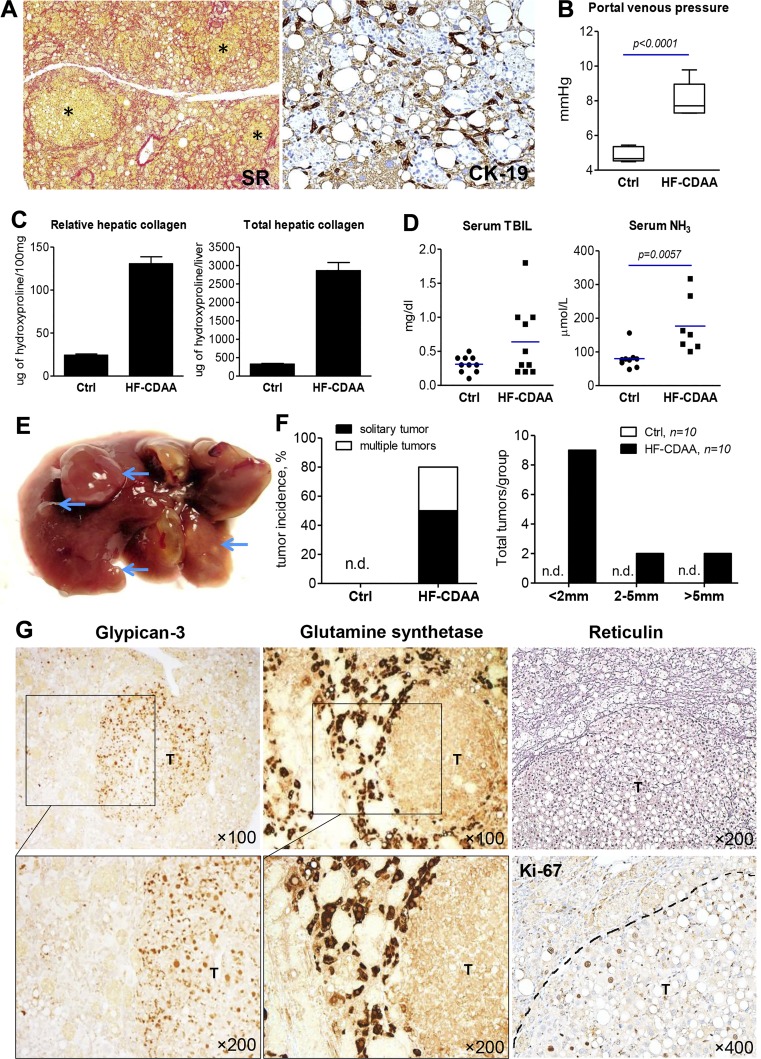
Development of cirrhosis and hepatocellular carcinomas (HCC) in mice on 24 wk of high-fat choline-deficient, amino acid-defined (HF-CDAA) feeding. C57Bl/6J mice were fed control (*n* = 10) or HF-CDAA diet (*n* = 10) ad libitum for 24 wk. *A*: representative histopathology: collagen staining [Sirius red (SR), *left*, ×50] shows severe perisinusoidal pan-lobular fibrosis with regenerative nodule formation (*), a massive ductular reaction [cytokeratin 19 (CK19 immunohistochemistry), *right*, ×200]. *B*: portal venous pressure, measured invasively at study end point. Box and whiskers plot (mean, minimum, and maximum values indicated). *C*: relative and total hepatic collagen content. *D*: serum total bilirubin (TBIL) and ammonia (NH_3_) levels. *E*: representative macroscopic image of HF-CDAA liver with tumors. Arrows indicate visible tumor-like lesions >1 mm in size. *F*: incidence and overall tumor burden in all mice receiving control or HF-CDAA diet for 24 wk. *G*: representative images of tumor (T) immunostainings for HCC markers glypican-3 and glutamine synthetase (*left* and *middle*; blow-up magnification shows tumor/nontumor interface). Note typical loss of pericellular reticulin staining pattern (*top right*) and increased cell proliferation within tumor (Ki-67 staining, *bottom right*, tumor border highlighted by dotted line). Data are means ± SE; *n* = 10 mice per group; n.d., not detected.

Finally, we assessed long-term disease progression by extending the feeding with the HF-CDAA diet to 24 wk. At the end of the study, body weight and relative liver and relative spleen weights significantly increased by 5% (*P* = 0.0432), 10% (*P* = 0.0305), and 27% (*P* = 0.0217) compared with mice fed the diet for 12 wk (Supplemental Table S11). Histologically, all HF-CDAA-fed mice developed signs of frank cirrhosis with severe perisinusoidal pan-lobular chicken wire fibrosis, regenerative nodule formation, and a pronounced ductular reaction (Fig. 7*A*). At study end point (24 wk), all HF-CDAA mice developed significant portal hypertension (8.12 ± 0.35 vs. 4.89 ± 0.19 mmHg in age-matched healthy controls, *P* < 0.0001), as assessed via direct (invasive) portal venous pressure measurement (Fig. 7*B*). Biochemically, total hepatic collagen content after 24 wk of HF-CDAA diet feeding increased up to ninefold vs. age-matched healthy controls, compared with 4-, 5.9-, and 7.8-fold, respectively, at 4, 8, and 12 wk, indicating that collagen deposition continued relentlessly and in a linear manner (Fig. 7*C* and Supplemental Table S11). Serum TBIL was increased twofold or more in 4 of 10 mice, and serum NH_3_ levels were significantly elevated (Fig. 7*D*) in HF-CDAA mice compared with age-matched healthy controls, indicating a loss of liver function, as a second clinically relevant end point. Macroscopic examination of livers demonstrated that 8 of 10 HF-CDAA-fed mice developed visible liver tumors (>1 mm), whereas none of sex- and age-matched controls on regular chow developed any lesions (Fig. 7*E*). Five of eight tumor-bearing animals developed a solitary neoplasm while another three had multiple tumors, with an overall 80% tumor incidence (8/10). The size of tumors ranged from 1 to 12 mm. Tumor burden was further characterized in HF-CDAA fed mice in terms of both tumor numbers and size (Fig. 7*F*). Thus, HE stainings of liver tumors were examined by an expert clinical pathologist (I. Nasser) and phenotyped via IHC for the HCC markers glypican-3 and GS, as well as reticulin stain and IHC for cell proliferation marker Ki-67. Based on histological assessment, including cytological characteristics (mitotic activity, anisokaryosis, cytoplasm inclusions) and expression of at least one of the HCC markers (glypican-3, GS, or both), 12 of 13 tumors were classified as well-differentiated HCC (Fig. 7*G*). HF-CDAA-induced hepatocellular neoplasms demonstrated a typical pattern of diminished reticulin staining (17) and increased rate of cell proliferation as assessed by Ki-67 immunostaining (Fig. 7*G*).

## DISCUSSION

Robust and progressive fibrosis, optimally with signs of liver decompensation and the development of HCC, is a critical requirement for animal models of NASH. However, these goals have been difficult or impossible to achieve, especially in widely used mouse models. Here, we performed a direct, quantitative comparison of several modifications of the MCD diet models in C57Bl/6J vs BALB/c mice. Among tested interventions, we identified the 60% fat HF-CDAA diet as an optimal inducer of steatohepatitis with robust fibrosis that rapidly progresses to cirrhosis, liver decompensation, and HCC. The remarkably extensive fibrosis in the HF-CDAA diet model is associated with a pronounced ductular reaction, has a limited spontaneous reversibility, and is driven by dietary fat in a dose-dependent manner. Moreover, when fed the HF-CDAA diet, the obesity-prone C57Bl/6J mice showed a more aggressive NASH than the fibrosis-prone BALB/c mice.

The lack of mouse models for NASH that develop robust liver fibrosis and related hard end points represents a significant challenge to the development of effective therapies for NASH. Absence of fibrosis in simple overfeeding models using HF diet prompted the addition of “second hits” such as high fructose or high cholesterol (or both) to induce liver injury and chronic inflammation. These efforts resulted in the development of several obesity-driven NASH models that more faithfully resemble metabolic aspects of human disease (2, 5). However, the rate of fibrosis progression achieved with these models is too slow to enable formal drug efficacy studies that target advanced fibrosis or cirrhosis and clinically relevant end points, such as liver failure and HCC. Asgharpour and Michael recently reported diet-induced NASH mouse model with significant fibrosis (DIAMOND and ALIOS); however, at least 36 wk was needed to reach the F2 fibrosis stage (20) and 52 wk to achieve the F3 fibrosis stage (2). On the other hand, the MCD diet does not recapitulate obesity, insulin resistance, and the metabolic syndrome, central features of human NASH but, due to disruption of phospholipid homeostasis, induces severe steatohepatitis with fibrosis apparent as early as 4 wk, but severe overall deterioration including dramatic loss of body weight (12). In contrast, the less disruptive CDAA diet leads to body weight gain, mild insulin resistance, and all histological features of human NASH including mild to moderate fibrosis (11). In the present study, we found that all groups fed MCD and CDAA diets with low or high fat for 8 wk developed varying degrees of histologically detectable perisinusoidal, chicken wire fibrosis characteristic of NASH. However, the 60% fat HF-CDAA stood out as by far the most fibrogenic (Figs. 1 and 2 and Supplemental Table S3). Strikingly, quantitative analysis of collagen accumulation using the “gold standard” hydroxyproline assay revealed a five- to sixfold increase in total liver collagen compared with normal controls (and an over twofold higher collagen content than the LF-CDAA group after 8 wk (*P* < 0.001; Fig. 1*F*). The profibrogenic ductular reaction, as assessed by CK19 staining, closely matched the extent of fibrosis and HSC activation in these settings (Fig. 2).

Feeding the CDAA diet with increasing fat content led to a dose-dependent increase in progressive fibrosis that was accompanied by a similar increase in ductular proliferation, identifying dietary fat as an important driver of fibrogenesis in the HF-CDAA model (Fig. 4). It is possible that the ductular reaction functionally drives fibrotic response in the HF-CDAA model, as we reported in biliary (29, 30, 33) and in advanced nonbiliary (hepatotoxin-induced) fibrosis (21). Importantly, the obesity-prone C57Bl/6J strain demonstrated greater alterations in hepatic lipid metabolism and was more susceptible to HF-CDAA-induced fibrosis than the obesity-resistant (but highly fibrosis-prone) BALB/c strain (Supplemental Fig. 3 and Supplemental Table S7), further supporting the notion that dietary/metabolic mechanisms are the major pathological drivers of fibrogenesis, inflammation, and, finally, cirrhosis, hepatic decompensation, and HCC in this model. Severe fibrosis in the HF-CDAA diet model was associated with specific, profound alterations in lipid metabolism-related genes encoding both isoforms of SCD1 and SCD2 (Fig. 5) which catalyze the conversion of long-chain fatty acids into monounsaturated fatty acids, a critical step in de novo triglyceride synthesis (27). SCDs catalyze a critical step in the production of active, lipid-modified Wnt proteins (38) and have been mechanistically implicated in liver fibrosis and cancer (22). Suppression of the SCD1 gene, which was downregulated by CDAA itself, and further suppressed by high fat, has been previously described in moderately fibrotic MCD diet-fed mice (39) and is unlikely to substantially account for severe fibrosis. On the other hand, the SCD2 gene, which is expressed in embryonic but not adult liver and is indispensable during development (25), was markedly and dose-dependently induced by dietary fat (Fig. 5). The regulation pattern of SCD2, previously identified as a direct downstream target gene of the transcription factor SREBP2 (16) was nearly superimposable on that of SREBP2 (but not SREBP1). In the context of available mechanistic evidence, these observations suggest that SREBP2, normally absent from healthy adult liver, is induced by the HF-CDAA diet and activates SCD2, which in turn mediates profibrogenic effects of the HF supplementation in our model.

Longitudinal analysis was not possible past 8 wk in MCD-fed mice due to excessive weight loss and emaciation. In contrast, HF-CDAA-fed mice maintained their body weight and did not develop any gross abnormalities when analyzed longitudinally for up to 24 wk. The HF-CDAA mice developed histological signs of steatohepatitis with elevated transaminases starting at the earliest time point tested (4 wk), persisting essentially unchanged until the latest studied time point (24 wk). Histological and biochemical analysis of collagen deposition suggested that fibrosis progressed relentlessly, with severe pan-lobular chicken wire perisinusoidal fibrosis (F3 CRN fibrosis stage) and an eightfold increase in hepatic collagen established already at 12 wk, reaching cirrhosis stage (F4), and a ninefold increase in hepatic collagen at 24 wk (Figs. 3 and 7). Quantitatively, this represents the fibrosis progression rate comparable to that observed in the genetic, biliary-type “rapid fibroser” BALB/c;*Mdr2^−/−^* mouse model, the most aggressive liver fibrosis progression reported so far in mouse species (17). Thus, relative hepatic collagen content in BALB/c;*Mdr2^−/−^* mice at 8 wk of age was 75.3 ± 6.8 μg/100 mg [total hydroxyproline 1,282 ± 69.2 μg/liver (17)] compared with 76.7 ± 6.5 (total hydroxyproline 1,464 ± 82.4 μg/liver; Fig. 1, *E* and *F*) and 64.3 ± 2.9 (total hydroxyproline 1,320 ± 77.3 μg/liver; Fig. 3, *B* and *C*) that we report here for 8 wk of HF-CDAA feeding in two independent experiments. Accordingly, dramatic differences in transcriptional fibrogenesis activity were observed in the HF-CDAA model, with key profibrogenic transcripts being overexpressed severalfold compared with HF-MCD diet-fed and strain-matched C57Bl/6J mice at *week 8* (Fig. 3*E* and Supplemental Fig. S1).

Even if advanced, liver fibrosis can reverse in animal models and in humans once the major stimuli of chronic hepatic inflammation are eliminated (34). However, we and others have reported earlier, that upon careful examination, the extent of experimental fibrosis reversibility varies greatly depending on underlying etiology, ranging from complete resolution with collagen degradation and removal in biliary type fibrosis (35) to incomplete reversal in postnecrotic fibrosis, with long-term persistence of collagen fibers within a reorganized fibrotic matrix (18, 31). Since there are few data on the reversibility of advanced fibrosis in experimental models of steatohepatitis, we investigated reversibility of HF-CDAA-induced fibrosis in detail. Histological and biochemical analysis of recovery from fibrotic NASH induced by 8 wk of HF-CDAA revealed persistence of excess collagen during 12-wk recovery, suggesting little “true” spontaneous reversibility, even at histological CRN stages F2–F3, despite complete reversibility of steatosis and inflammation. However, fibrotic scars underwent dramatic remodeling (splitting and reorganization of collagen bands around *week 4* of reversal), but the collagen fibers remained readily detectable throughout the liver parenchyma even after 12 wk of recovery, with no significant reduction in overall collagen content (Fig. 6). We and others (18, 23, 36) have previously demonstrated that fibrotic extracellular matrix (ECM) stabilization via collagen cross-linking plays a central role in limiting reversibility of hepatic fibrosis. In the HF-CDAA diet model studied in the current study, poor reversibility was associated with a high degree of collagen cross-linking at PF (>18% at 8 wk on HF-CDAA diet), which is comparable to that achieved with the model using escalating doses of CCl4 in the same strain [25% after 12 wk of repeated CCl4 injections vs. 4.5% in normal livers (36)]. In contrast to recovery from CCl4, collagen cross-linking appears to continue for at least 4 wk after cessation of pathogenic stimuli in the HF-CDAA diet model, as demonstrated by a significant increase of the highly cross-linked (insoluble) fraction during recovery (Fig. 6*D*). Interestingly, the insoluble collagen fraction markedly decreased by ~50% by *week 12* of recovery (Fig. 6*D*), whereas the average total collagen content showed only a trend to decline (*P* = 0.186, NS; Fig. 6*C*). It is tempting to speculate that the 12-wk time point represents an early stage of fibrosis reversal, preceded by a breakdown of the highly cross-linked ECM, an interesting area for further mechanistic studies. This limited spontaneous fibrosis reversal makes the HF-CDAA model highly suitable to test direct “fibrolytic” approaches aiming at pharmacological reversal of fibrosis as well as inhibitors of collagen cross-linking.

Another relevant finding is that within only 24 wk the HF-CDAA-induced steatohepatitis progressed to clinically relevant end points such as development of cirrhosis, portal hypertension, and signs of liver function failure (significantly increased serum bilirubin and NH_3_), and primary liver cancer (Fig. 7). To our knowledge, this represent the fastest (clinically relevant) disease progression observed in mouse species that were once believed to be cirrhosis resistant (4). HCC in NASH, a growing concern, has a biology distinct from HCC due to other etiologies. Thus, an estimated 30–40% of NASH HCC arises in noncirrhotic (but often more advanced fibrotic) livers, likely driven by the cancerogenic effect of fatty liver inflammation and progenitor cell proliferation that are also hallmarks of our HF-CDAA model. Notably, tumors induced by 24 wk of HF-CDAA feeding aberrantly expressed glypican-3 and/or GS, phenotypically resembling human HCC. Several recent studies reported development of mouse models of NASH-associated HCC (26). However, except for a model of diethylnitrosamine-induced chemical carcinogenesis in HF diet-fed mice, HCC develops very late (from 52 wk on) on the respective diet, with varying penetrance, making these models impractical for anti-cancer drug screening (or for long-term evaluation of antifibrotic therapies). Thus, at 12 mo, 40% of ALIOS mice (8) and 89% DIAMOND mice (2) developed HCC. To date, only one model that utilized low-CCl4 as a nonnutritional and highly toxic “accelerator” of Western diet-induced NASH, reported a 100% HCC incidence at 24 wk (47).

One weakness of the HF-CDAA model must also be noted, specifically that the mice do not develop obesity and only mild insulin resistance (7). The need for overfeeding as a means to achieve pathogenetic similarities to human NASH was clearly identified in comparative genome profiling studies (46). However, we demonstrate that high saturated/monounsaturated dietary fat as a “second hit” activates SREBP/SCD genes and directly drives disease pathology in the CDAA model, importantly without the need to add supraphysiological levels of cholesterol to the diet (11). Contrary to what has been widely assumed, and based on lack of an overt obesity phenotype in the CDAA model (13), our data strongly suggest that fat overfeeding appears to be essential for the severe fibrotic NASH phenotype. Our finding that strain-specific differences in liver fibrosis (C57Bl/6J > BALB/c) parallel the magnitude of changes in lipid metabolism genes induced by HF-CDAA diet rather than genetic susceptibility to fibrosis per se (BALB/c > C57Bl/6J) further corroborates this notion (Fig. 5, Supplemental Fig. S1, and Supplemental Table S7). Importantly, there is evidence of choline deficiency and a phospholipid dysbalance playing a role in (at least a subset of) human NASH (44), further supporting the relevance of the HF-CDAA intervention as a simple, practical, and predictive NASH model that leads to the end points deemed relevant for approval by the regulatory authorities for new drug therapies in NASH (42). Finally, our study provides a comprehensive, longitudinal characterization of disease evolution in the HF-CDAA model as well as optimized means to quantitatively assess fibrosis, cirrhosis, liver function, and HCC as clinically relevant end points for future basic research and preclinical drug testing.

A limitation of our study is the lack of assessment of the potential key factors of the development of human NAFLD other than fibrosis, such as alteration in insulin signaling, intestinal barrier function, and/or Toll-like receptor-dependent signaling. Also, the functional significance of the SREBP/SCD axis and underlying molecular mechanisms remain to be elucidated.

In conclusion, HF-CDAA-fed C57Bl/6J mice demonstrate steatohepatitis with dietary fat-driven dysregulation of lipid metabolism genes, a pronounced ductular reaction, robust and rapidly progressive fibrosis, and clinically relevant sequelae, such as development of cirrhosis, portal hypertension, partial loss of liver function, and HCC by 24 wk. We have further shown that the fibrotic response is driven by dietary fat and is poorly reversible once established. This represents a robust and practical preclinical tool that should permit formal testing of drug candidates that address the clinically relevant, liver related end points of progressive steatohepatitis and/or induce reversal of advanced NASH-induced fibrosis.

## GRANTS

This study was in part supported by a research grant from PSC Partners Seeking a Cure Canada, and an institutional grant from the Department of Medicine (Beth Israel Deaconess Medical Center) to Y. V. Popov. D. Schuppan is funded by European Union Horizon 2020 under grant agreement no. 634413 (EPoS, European Project on Steatohepatitis) and 777377 (LITMUS, Liver Investigation on Marker Utility in Steatohepatitis), and by German Research Foundation collaborative research project grants DFG CRC 1066/B3 and CRC 1292/08.

## DISCLOSURES

No conflicts of interest, financial or otherwise, are declared by the authors.

## AUTHOR CONTRIBUTIONS

G.W. and Y.P. conceived and designed research; Y.P. secured the funding and supervised the study; G.W., P.A., K.V., P.H., L.T., and S.Z. performed experiments; G.W., P.A., I.N., P.H., L.T., S.Z., and Y.P. analyzed data; G.W., I.N., D.S., and Y.P. interpreted results of experiments; G.W. and Y.P. prepared figures; G.W. and Y.P. drafted manuscript; G.W., D.S., and Y.P. edited and revised manuscript; G.W., P.A., K.V., I.N., P.H., L.T., S.Z., D.S., and Y.P. approved final version of manuscript.
